# Push Screw Technique: A Technical Trick to Optimize Coronal Reduction of Distal Femur Fractures

**DOI:** 10.7759/cureus.92143

**Published:** 2025-09-12

**Authors:** Alex Moses, Cameron Foster, Porter Young

**Affiliations:** 1 Orthopedic Surgery, University of Florida College of Medicine-Jacksonville, Jacksonville, USA

**Keywords:** comminuted distal femur fracture, complex distal femur fracture, distal femur fracture, distal femur locking plate, nonunion of distal femur

## Abstract

The "push screw" technique is an adjunct for managing coronal alignment in distal femur fractures fixed with lateral locking plates. Distal femur fractures can be challenging to reduce due to technical challenges in plate placement, leading to varus malalignment and functional consequences. The “push screw” technique involves using a short locking screw proximal to metaphyseal comminution to medialize the proximal shaft, thereby improving coronal alignment. This article presents a case series of distal femur fractures in which the technique was successfully utilized. The "push screw" technique offers a viable option to avoid the golf club deformity associated with lateral plate fixation, emphasizing its safety, ease of implementation, and effectiveness in achieving and maintaining fracture reduction.

## Introduction

The use of lateral locking plates for fixation of distal femur fractures is still commonplace, despite the increased use of other treatment modalities such as intramedullary nailing. In fact, many surgeons still consider lateral locked plating to be the gold standard for the management of distal femur fractures, especially for fractures with complex articular involvement [1-4]. However, applying these plates can be technically challenging and sometimes can lead to residual deformity. One of the most common and well-described pitfalls that can occur while using lateral locked plating is the “golf club” deformity [5]. The pre-contoured nature of the plate is typically designed so that the distal aspect of the plate rests on the anterolateral surface of the distal femur. Due to the trapezoidal nature of the distal femur in the axial plane, the anterolateral surface is more medial than the posterior articular block [6,7]. For this reason, when the distal plate is placed too posteriorly, it is also too lateral, and the plate over-medializes the articular block when securing the proximal plate to the proximal bone [1,8]. A similar deformity can occur in patients who have a more bulbous metaphysis compared to the shaft [9]. Clinically, this can result in varus malalignment, as well as rotational deformity, which can manifest as gait alterations, osteoarthritis, and has been shown to increase the risk of fracture nonunion [4-6]. 

To avoid this pitfall, various techniques have been employed over the years. One technique requires intermittent fluoroscopic images while fixing the plate to the proximal shaft after securing the distal block. Using non-locking cortical screws to pull the proximal plate to the bone, the surgeon must keenly recognize when proper alignment has been obtained, even if it requires stopping non-locking screw advancement. If the plate is not flush with the proximal bone at that time, the technique requires using locking screws for the rest of the screws proximally, allowing the plate to remain stable while not having bony contact, creating an “internal external fixator” [1]. While this can be effective, the plate is more prominent, which could irritate the patient’s iliotibial band, resulting in persistent postoperative pain. Another technique involves “shimming” a plate where washers are placed between the plate-bone interference to avoid over-lateralization of the proximal femoral shaft segment [10]. Inverting intraoperative contralateral extremity radiographs has been used as a template for reduction of the injured extremity, to reproduce the patient’s presumed normal anatomy [11]. Dual plating can also be utilized to balance coronal alignment. This technique requires using the medial plate to buttress the distal block back laterally, fighting the medializing forces of the lateral plate. Medial plating can be effective; however, it requires a second approach and additional hardware [12]. The “push screw” technique described in this article can be applied when using lateral locked plating and avoids additional exposure. It is most advantageous for obtaining a reduction in the coronal plane in the setting of metaphyseal comminution, bone loss, or a mismatch in femur plate anatomy.

## Technical report

**Figure 1:**
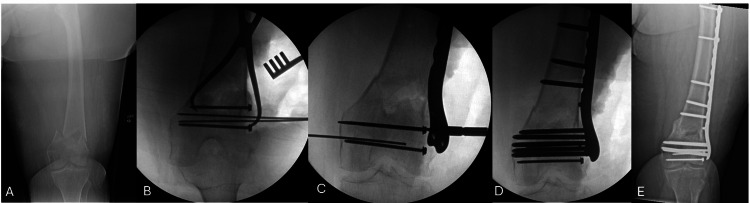
Left distal femur fracture in a 71-year-old female AP image of a left distal femur fracture in a 71-year-old female after a ground-level fall (A). Compression with a lag screw across the articular fracture lines (B), followed by bridge plating of the metaphyseal comminution using the bridge plating technique, with concern for medialization of the articular block (C). A push screw is used to medialize the femoral shaft into appropriate coronal alignment (D). At 5.5-month follow-up, the fracture is healed with maintained alignment and a lateral distal femoral angle of 79.6° (E).

The “push screw” technique was designed as a modification of the standard technique to apply a lateral locked plate. The patient should be placed supine on a radiolucent flat top table with an ipsilateral hip bump. The distal articular block can be approached using a standard lateral approach to the distal femur or a lateral parapatellar approach. Any intra-articular fractures are then anatomically reduced and compressed with lag screw fixation to restore the distal articular block (Figure 1A, 1B). Next, the lateral locked plate is properly positioned on the anterolateral distal femur and provisionally fixed with K-wires or locking screws to the articular block. The articular block is then reduced back to the intact shaft via indirect reduction techniques. While assessing the reduction in the setting of metaphyseal comminution, the shaft will frequently lateralize to the plate proximally without placing any screws, thus medializing the articular block. This occurs because of the lack of a metaphyseal buttress and soft tissue compression of the vastus lateralis to the plate (Figure 1C). The “push screw” can be utilized to medialize the shaft back into proper coronal alignment. The “push screw,” which is a short locking screw, is introduced proximal to the metaphyseal comminution and is inserted into the plate without drilling. This translates the shaft medially and obtains the reduction once the screw locks into the plate (Figure 1D, 1E). “Push screws” are typically 5.0 mm in diameter and (10-14) mm in length. Periprosthetic screws with blunt screw tips work best to optimize the surface area and avoid cortical perforation in poor bone quality. Once the reduction is confirmed to be appropriate radiographically, the remaining shaft screws are inserted in the standard fashion, depending on the plate’s position relative to the bone and the bone quality. The “push screw” is left in place with the final construct. The main advantage of this screw is obtaining and maintaining a quality reduction in the coronal plane in the setting of metaphyseal comminution and bone loss.

It should be noted that this technique does not address rotational or sagittal alignment. Additionally, the amount of medialization of the proximal shaft is dependent on the length of the push screw. Assessing the proper length of the “push screw” can require some trial and error, but this can be easily changed to optimize the reduction. Moreover, one must be careful to assess the competency of the lateral wall. If the bone is osteoporotic or too close to the fracture site, it is possible to either break off the lateral cortex or sink the screw into the lateral cortex despite there being no drill hole.

**Figure 2:**
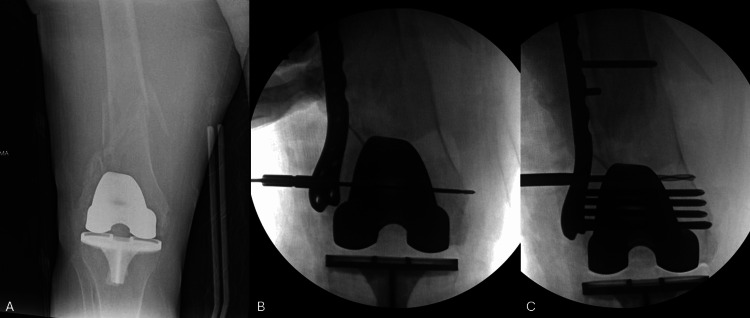
A 76-year-old female with a periprosthetic right distal femur fracture AP image of a 76-year-old female with a periprosthetic right distal femur fracture after a ground-level fall (A). Intraoperative fluoroscopic images demonstrating medialization of the articular block (B), which is improved with use of a push screw (C).

**Figure 3:**
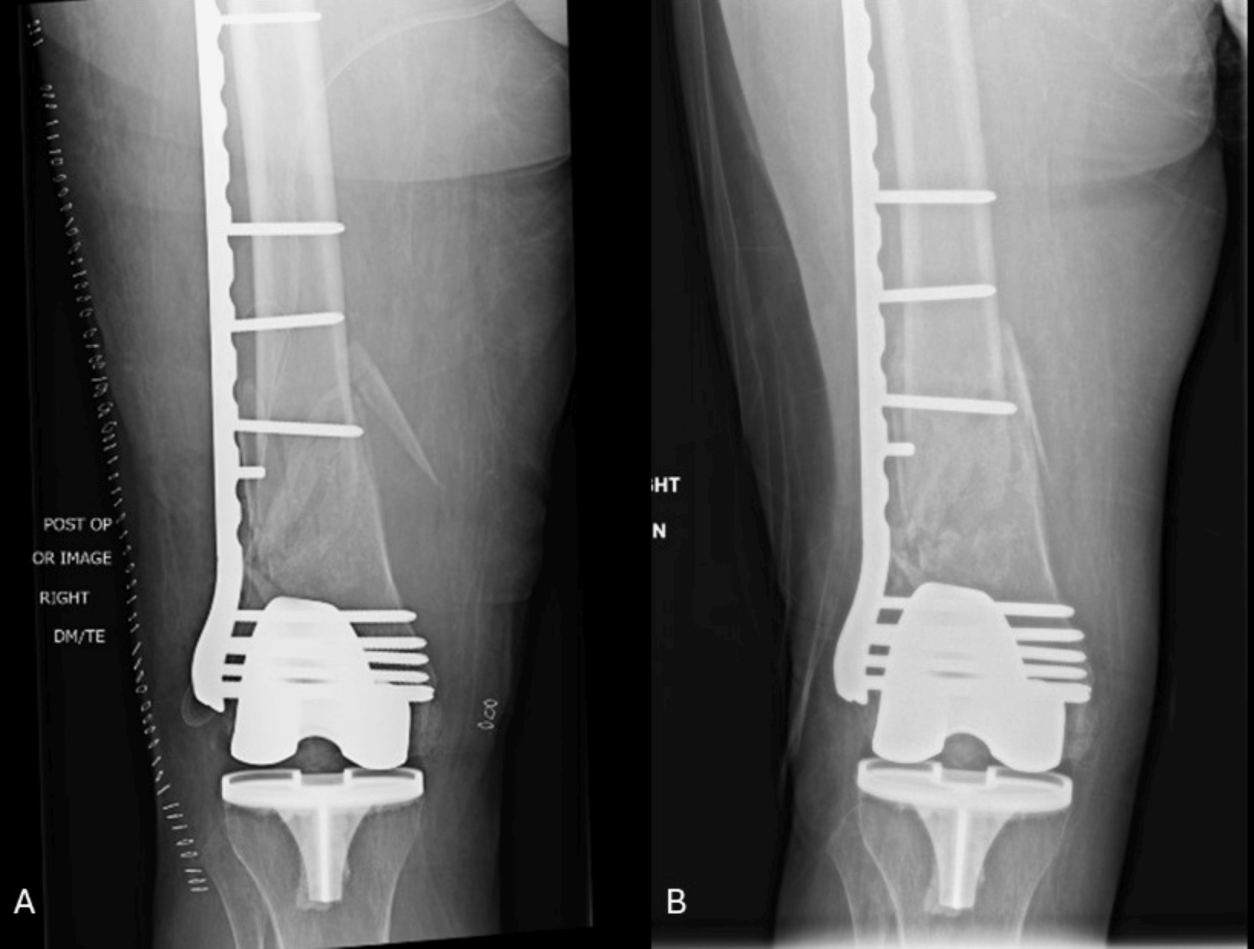
Postoperative and follow-up radiographs Immediate postoperative AP of the distal femur (A) and 4.5-month follow-up (B) demonstrating radiographic signs of healing and a lateral distal femoral angle of 82.9°.

The following cases highlight the clinical scenarios where employing the “push screw” can be advantageous. The first case involves a 76-year-old female who sustained a comminuted right periprosthetic distal femur fracture after a ground-level fall (Figure 2A). Intraoperatively, during reduction utilizing the previously mentioned technique, the articular block was noted to be medializing (Figure 2B), and the “push screw” was utilized to improve coronal alignment and avoid a golf club deformity (Figure 2C). She was made non-weight-bearing for 12 weeks due to the extensive metaphyseal comminution and poor bone quality. At her most recent follow-up, 4.5 months post-op, she was ambulating with a walker and showed radiographic evidence of healing (Figure 3).

**Figure 4:**
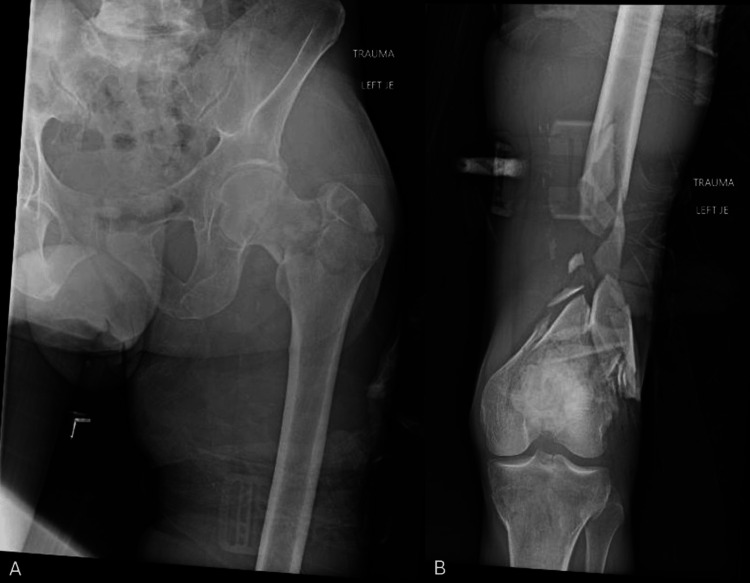
Distal femur fracture with segmental bone loss AP proximal femur (A) and AP distal femur (B) radiographs demonstrating a comminuted distal femur fracture with associated bone loss and an ipsilateral intertrochanteric femur fracture.

**Figure 5:**
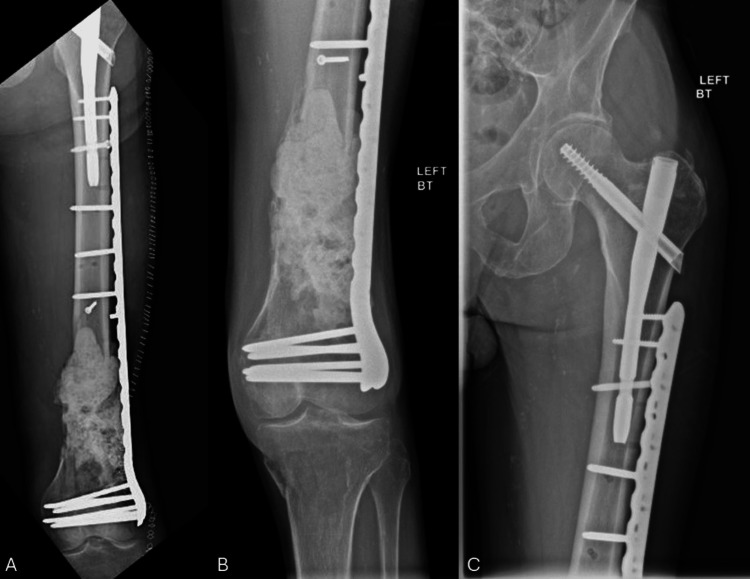
Postoperative and follow-up radiographs Immediate postoperative AP of the left femur after use of a push screw to optimize coronal alignment in the first stage of the induced membrane technique (A). Eight-month follow-up demonstrating maintained alignment (lateral distal femoral angle: 82.4°) and healing of the intertrochanteric femur fracture (B, C).

The next case is a 71-year-old male involved in a motor vehicle collision where he sustained a Gustilo-Anderson type IIIA open left distal femur fracture and an ipsilateral intertrochanteric femur fracture (Figure 4A, 4B). The intertrochanteric femur fracture was treated with a short cephalomedullary nail, and the distal femur fracture was fixed with a lateral plate utilizing the “push screw” technique. The patient was left with a large bone void due to the open injury and debridement of nonviable bone. Therefore, he underwent the first stage of an induced membrane technique with antibiotic cement spacer placement. The “push screw” was utilized to manage the coronal alignment in the setting of extensive metaphyseal bone loss (Figure 5A). He was last seen at 12 months postop and declined to undergo the second stage of the induced membrane technique for personal reasons. However, he remained ambulatory with a walker and was independent with activities of daily living (Figure 5B, 5C).

## Discussion

The "push screw" technique shows promise as a reproducible method for managing intraoperative coronal alignment when utilizing lateral plating for distal femur fractures. Using periprosthetic locking screws just proximal to the fracture site helps medialize the proximal shaft, ultimately improving coronal alignment and minimizing the risk of the golf club deformity. It is minimally invasive, cost-effective, and technically straightforward. The screw is produced by most vendors that manufacture lateral locked plates, making implementation readily available. However, determining the correct screw size may require some trial and error. Additional pitfalls include the potential for propagating a lateral cortex fracture at the site where the screw engages the bone, especially in cases of osteoporosis or compromised bone due to distal fracture extension. Another downside of the technique is that surgeons often restrict weight-bearing when utilizing lateral locked plating, compared to dual constructs such as nail-plate or dual-plate constructs [13,14].

## Conclusions

In conclusion, the “push screw” technique is a valuable and simple adjunct when managing coronal alignment in distal femur fractures treated with lateral locking plates. It is most effective in cases of metaphyseal comminution, where the risk of translational malreduction is high. Achieving proper reduction in distal femur fractures is critical to decreasing the risk of malunion, nonunion, and poor functional outcomes. The “push screw” technique is a promising reduction tool, and future studies should aim to objectively measure its effectiveness in greater depth than is possible with this case series.
